# Progress in the development of gelling agents for improved culturability of microorganisms

**DOI:** 10.3389/fmicb.2015.00698

**Published:** 2015-07-23

**Authors:** Nabajit Das, Naveen Tripathi, Srijoni Basu, Chandra Bose, Susmit Maitra, Sukant Khurana

**Affiliations:** ^1^Department of Biological Sciences, Indian Institute of Science Education and Research KolkataKolkata, India; ^2^School of Biotechnology, Kalinga Institute of Industrial Technology UniversityBhubaneswar, India

**Keywords:** agar, gelatin, xanthan gum, guar gum, gellan gum, isabgol, carrageenan, katira gum

## Abstract

Gelling agents are required for formulating both solid and semisolid media, vital for the isolation of microorganisms. Gelatin was the first gelling agent to be discovered but it soon paved the way for agar, which has far superior material qualities. Source depletion, issues with polymerase-chain-reaction and inability to sustain extermophiles etc., necessitate the need of other gelling agents. Many new gelling agents, such as xantham gum, gellan gum, carrageenan, isubgol, and guar gum have been formulated, raising the hopes for the growth of previously unculturable microorganisms. We evaluate the progress in the development of gelling agents, with the hope that our synthesis would help accelerate research in the field.

## Introduction

Gelling agents are added to the liquid microbial media to convert them into semi-solid or solid media. Generally some colloidal polysaccharides and certain proteins of microbial and plant origin act as solidifiers or stabilizers in the medium by forming continuous three-dimensional molecular network. Gelling agents provide firmness to the medium and influence its diffusion characteristics. Diffusion rate is dependent on the viscosity of the medium, which subsequently depends on the concentration and physicochemical characteristics of the agent (Ackers and Steere, 1962; Palaniraj and Jayaraman, 2011). Certain gelling agents can reverse between liquid and gel state depending on the temperature, a property that adds much to their desirability. A good solidifier tends to be colorless, odorless and a good retainer of moisture.

Several mesophiles and extremophiles are currently unculturable or poorly culturable due to the lack of proper biotic and abiotic factors. Each gelling agent has a limited range of pH and temperature of optimal functioning and different gelling agents can be degraded by a different set of microorganisms, necessitating the need of several gelling agents. In recent times, traditional sources of gelling agents are being overused, further necessitating the need of new gelling agents.

Recent work on gelling agents that can withstand variety of temperatures and pressures have helped in culturing newer microbes, including few extremophiles, which could otherwise not be grown (Becker et al., 1998; Jain et al., 2005). Despite important applications in microbiology, there is a lack of synthesis of information about major gelling agents at one place. We attempt to fill that lacuna by covering gelling agents, which support the growth of mesophiles and also agents, which support the growth of extremophiles.

**Figure 1:**
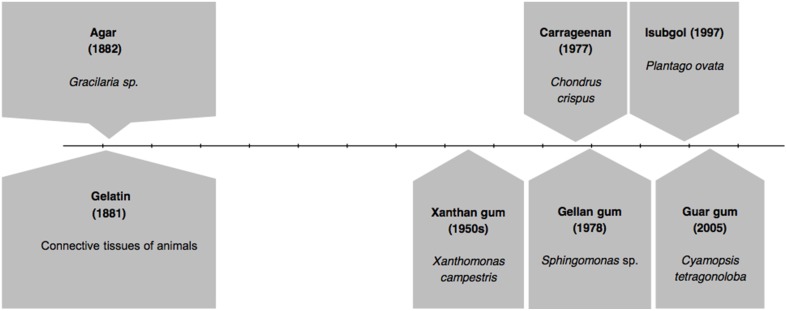
**Timeline of discovery of various gelling agents**.

In Figure 1 we present the timeline of discovery of first gelling agents: gelatin and agar, followed by subsequent important developments. After several decades of the discovery of agar and gelatin, in 1950s, another alternative agent, Xanthan gum was reported to be of use. It was derived from a bacterium called *Xanthomonas campestris* (García-Ochoa et al., 2000; Bellini and Caliari, 2014). Two other gelling agents came in two successive years: carrageenan in 1977 (Lines, 1977; Jagdale and Pawar, 2014; Kuo et al., 2014; Razavi et al., 2014) and gellan gum in 1978 (Nampoothiri et al., 2003; Jamshidian et al., 2014). Isubgol was proposed as another alternative in the year 1997 (Jansson et al., 1983; Jain et al., 1997; Harding et al., 2004; Fialho et al., 2008), which was followed by the latest of its kind guar gum in 2005 (Jain et al., 2005; Kirchmajer et al., 2014). Figure 1 also represents the source of the major gelling agents.

## Gelatin and agar: the first gelling agents

Solid media, which is achieved by the addition of gelling agents to the liquid broth, is more suited for the separation and isolation of microorganisms than liquid media. Gelatin was used to obtain the first solid media in 1881 by Robert Koch (Poppe, 1997; Jain, 2011; Petrovski and Tillett, 2012). Its digestion by bacteria and melting temperature at 37°C limited its use. These problems associated with gelatin propelled the search for alternative agents. The use of agar as an alternative to gelatin was first proposed by Angelina Hesse the wife of Walther Hesse, an associate of Koch in 1882 to prepare solid culture (McLachlan, 1985; Becker et al., 1998).

Various advantages of agar over gelatin made it popular, as it is stable over a wide range of temperature (solidification temperature between 32 and 42°C and melting temperature around 85°C) and thus is suitable for the growth of mesophilic organisms. The firmness of media increases directly in proportion to the concentration of agar (Kang et al., 1982; Giavasis et al., 2000; Sá-Correia et al., 2002; Nampoothiri et al., 2003; Jain, 2011). Additionally agar has good diffusion characteristics (Manjanna et al., 2010; Petrovski and Tillett, 2012) and has good clarity, low adhesiveness and is metabolically inert (McLachlan, 1985; Henderson and Kinnersley, 1988; Petrovski and Tillett, 2012). Agarases are a class of enzymes that are capable of degrading agar. A number of agarase-producing microorganisms, present mainly in the marine environments have been reported to degrade and utilize agar (Stanier, 1941; Ohta et al., 2004; Bannikova et al., 2008; Miyazaki et al., 2008). A novel agar-degrading bacterium, designated as strain KA5–BT has also been isolated from the soil (Sakai et al., 2014). The β-(1→4) linkage of agarose is hydrolyzed by most agarases yielding oligosaccharides. In *Pseudoalteromonas atlantica* (Day and Yaphe, 1975; Groleau and Yaphe, 1977), a well characterized agarolytic system the extracellular endo-β-agarase I depolymerizes agarose to neoagarotetraose.

Extensive usage of agar in laboratories has affected its natural sources, such as *Gelidium* sp., *Gracillaria* sp., and *Pterocladia* sp. (Lin and Casida, 1984; McLachlan, 1985; Schmidt and Rath, 2003; Kirchmajer et al., 2014) and increased its cost. Food grade agar has also been reported as a low-cost alternative in the preparation of solid microbiological media (Harding et al., 2004; Fialho et al., 2008; Petrovski and Tillett, 2012). The food grade agar is comparable to bacteriological agar in terms of its gelling and stability properties. However, the media produced with food grade agar have less clarity than that produced with bacteriological agar (Kang et al., 1982; Petrovski and Tillett, 2012).

## Development of alternative gelling agents

Many alternative gelling agents were discovered with time, such as carrageenan (Lines, 1977), kappa-carrageenan (Abbott and Chapman, 1981; Morris et al., 2012), gellan gum (Kang et al., 1982; Shungu et al., 1983; Kuo et al., 2014), isubgol (Shungu et al., 1983; Lin and Casida, 1984; Harris, 1985; Marteinsson et al., 1997; Babbar and Jain, 1998; Sahay, 1999; Atici et al., 2008; Ozel et al., 2008), guar gum (Shimomura and Kamada, 1986; Huang et al., 1995; Shigeta et al., 1996; Chen et al., 1999; Jain et al., 2005) and xanthan gum (Giavasis et al., 2000; Babbar and Jain, 2006; Prajapati et al., 2013). The building blocks and properties of these agents are presented in Table 1. Gelling agents have several applications in animal and plant cell culture, pharmaceutical and food industry and we expect cross fertilization from these fields for the development of novel gelling agents needed in microbial media.

**Table 1:** **Properties of various gelling agents**.

**Agent name**	**Building components**	**Characteristic feature**
Gelatin	Glycine, proline, hydroxyproline	Melts at 37°C; Stable over a narrow temperature range; Digestible by several bacteria
Agar	Linear polysaccharide of agarose and agaropectin	Melts at 85°C and thus used for growing mesophiles; Stable over a wide temperature range; No toxic bacterial inhibitors; Forms a clear gel
Gellan gum	A tetrasachharide of two D-Glucose, L-rhamnose, D glucoronic acid	Melts at 110°C and can be used for growing thermophiles; Forms stable gel at very low concentration; Forms a gel of higher clarity as compared to other agents
Xanthan gum	Pentasaccharide of two glucose, two mannose and glucoronic Acid	Melts at 270°C and can be used for growing various fungi and bacteria; Stable over a wide range of temperature and pH
Guar gum	Galactomannan (galactose and mannose)	Melts at 220°C and can be used for growing various fungi and bacteria; Highly soluble; High viscosity restricts its use; Degradable at lower pH; Poor clarity due to presence of impurities
Isubgol	Xylose, arabinose, galacturonic acid and traces of rhamnose and galactose	Melts at temperature >100°C; Stable in gel form; No cracking or drying problems; Forms gel even in cold water
Carrageenan	Alternate units of d-galactose and 3, 6-anhydro-galactose joined by a-1, 3 and B-1,4-glycosidic linkage.	Melts around a temperature range of 50–80°C; Suitable for the growth of alkaliphiles as remains stable even in high pH value

## Xanthan gum

Xanthan gum, a water soluble pentasaccharide produced by the fermentation of carbon sources, using plant-pathogenic bacterium *Xanthomonas campestris* (Paul et al., 1986; Kubo, 2003; Palaniraj and Jayaraman, 2011), consists of D-glucosyl, D-mannosyl and D-glucuronyl acid residues (Lines, 1977; Becker et al., 1998; Datta et al., 2011). Prosthecate bacteria *Verrucomicrobium* sp. GD, which has been isolated from activated sludge degrades xanthan (Muchová et al., 2009). Red pigmented gram positive bacteria can also degrade xanthan (Kennedy and Sutherland, 1994). Salt tolerant bacteria especially *Bacillus* species produces an inducible enzyme having extracellular xanthan degrading activity (Hou et al., 1986). Xanthan degradation leads to products like glucose, glucoronic acid, mannose, pyruvate mannose, acetylated mannose, and unidentified oligosaccharide and polysaccharide (Cadmus et al., 1982). Xanthan gum on its own does not form good gels but the gelling efficacy of Xanthan gum increases in combination with agar (McLean and Williamson, 1979; Greer and Yaphe, 1984; Barbeyron et al., 1998, 2000; Jain, 2011). This can drive decrease in agar usage and hence lower costs.

Due to its soft texture, xanthan gum is widely used as a thickener or viscosifier in both food and non-food industries. It also works as a stabilizer for a wide variety of suspensions, emulsions and foams (Becker et al., 1998; Michel et al., 2003). Xanthan gum, in combination with chitosan membranes, is used in the treatment of dermo-epidermal wounds (Michel et al., 2003; Bellini and Caliari, 2014). Various reports suggest its wide range of applications in pharmaceuticals (Gardin and Pauss, 2001; Jagdale and Pawar, 2014; Kuo et al., 2014; Razavi et al., 2014) and cosmetic industries (Jianlong and Yi, 1999; Jamshidian et al., 2014).

## Gellan gum

Gellan gum, a water soluble exo-polysaccharide produced by the bacterium *Sphingomonas elodea* (Jansson et al., 1983; Jain et al., 1997; Sahay, 1999; Harding et al., 2004; Fialho et al., 2008; Ozel et al., 2008), forms clear gels in the presence of multivalent cations (Jain, 2011; Kirchmajer et al., 2014). Industrially, it is inducibly produced by different strains of *Sphingomonas paucimobilis* (Kang et al., 1982; Giavasis et al., 2000; Sá-Correia et al., 2002; Nampoothiri et al., 2003; Sharma and Mazumder, 2014). Production of gellan gum by bacteria depends on several factors such as temperature, pH, stirring rate, oxygen transfer and composition of the fermentive medium. Gellan gum can be of few types that includes deacetylated, clarified and native gums (Manjanna et al., 2010) and is sold under different trade names such as Gelrite and Kelcogel (Lin and Casida, 1984; Schmidt and Rath, 2003; Kirchmajer et al., 2014). In its native form, it is a linear anionic exopolysaccharide composed of a tetrasaccharide repeat unit of two molecules of D-glucose, one of L-rhamnose and one of D-glucuronic acid (Harding et al., 2004; Fialho et al., 2008). It gels faster and of higher clarity as compared to agar (Kang et al., 1982). Stronger gels of gellan gum are formed if cations are present during solution to gel transition (Morris et al., 2012). *Paenibacillus* sp. isolated from activated sludge degrades gellan (Muchová et al., 2009). Red pigmented gram positive bacteria can also degrade gellan (Kennedy and Sutherland, 1994). High thermal stability of gellan gum makes it an ideal medium for growth of several thermophiles, such as thermophilic *Bacillus* sp., *Methanobacterium* sp., and *Methanobrevibacter* sp., and bacteria belonging to the genus *Thermotogales* (Shungu et al., 1983; Lin and Casida, 1984; Harris, 1985; Marteinsson et al., 1997).

In plant tissue culture media, gellan gum is used as a substitute to agar (Shimomura and Kamada, 1986; Huang et al., 1995; Shigeta et al., 1996; Chen et al., 1999). Gellan gum has been reported to have potential applications in the production of capsules, films and fibers, as well as dental and personal care products (Giavasis et al., 2000; Prajapati et al., 2013). Gellan gum is also used for microencapsulation, cell immobilization and in controlled drug release (Paul et al., 1986; Kubo, 2003).

## Carrageenan

Carrageenan, a gelatinous hydrocolloid extracted from the cell wall of marine algae *Chondrus crispus*, acts as a gelling substitute for agar in bacteriological media, especially the K salt of carrageenan (Lines, 1977; Datta et al., 2011). Carrageenans come in various molecular forms (McLean and Williamson, 1979; Greer and Yaphe, 1984; Barbeyron et al., 1998, 2000). Carrageenans are made up of repeating units of d-galactose residues. The connecting link between two d-galactose consists of alternate alpha(1→3) and beta(1→4) linkages (Michel et al., 2003). Both, kappa- and iota-carrageenan chains can adopt ordered conformations, which leads to the formation of crystalline fibers composed of aggregates of double-stranded helices (Michel et al., 2003). *Pseudoalteromonas carrageenovora* produce enzymes for the hydrolysis of iota, Kappa and lambda carrageenan by the breakage of β-1,4-linkage (Henares et al., 2010). ι-carrageenan specific extracellular carrageenase (Greer and Yaphe, 1984) is produced by *Alteromonas fortis*. *Z. galactanivorans*, a flavobacteria isolated from the red alga *Delesseria sanguinea* in Roscoff (Potin et al., 1991), secretes one κ-carrageenase (Potin et al., 1991) and one ι-carrageenase (Barbeyron et al., 2000). All the known carrageenases specifically cleave the β-(1→4) linkage of their respective substrates. A combination of kappa-carrageenan and gelatin has also been found to support the co-immobilization of aerobic and anaerobic bacteria (Gardin and Pauss, 2001).

The development of chitosan-carrageenan nanoparticles and polymers of alginate and carrageenan have potential applications in tissue engineering and regenerative medicine (Jianlong and Yi, 1999).

## Isubgol

Psyllium (Isubgol), a colloidal polysaccharide successfully used as an alternative gelling agent in tissue culture media has also been used in microbial culturing (Jain et al., 1997; Sahay, 1999; Ozel et al., 2008). It remains highly viscous at high temperatures and thus poses a problem in the adjustment of pH and the dispensing of media to the culture vessel (Jain, 2011). Studies have revealed that Isubgol husk, along with sodium alginate, can be formulated into glidazide loaded microparticles, which can regulate blood glucose level in diabetic animal models (Sharma and Mazumder, 2014).

## Guar gum

Guar gum, a polysaccharide of galactose and mannose, obtained from the endosperms of an annual leguminous plant *Cyamopsis tetragonoloba*, is a biodegradable exo-polysaccharide, which doesn't cause harm to the environment after its disposal (Jain et al., 2005). Guar gum is fermented by a gram-negative obligate anaerobe *Bacteroides ovatus* present in the human colon (Tomlin et al., 1988). Two galactomannanases and one α-galactosidase known as α-galactosidase I are involved in the breakdown of guar gum by *Bacteroides ovatus*. The two galactomannanases cleave the β-mannan backbone of guar gum into large pieces whereas α-galactosidase I removes galactose branches from the galactomannan segments. The backbone is further hydrolyzed by galactomannanases into single mannose residues (Valentine and Salyers, 1992). Extracellular enzymes of one strain of *B.variabilis* and one strain of *B.uniformis* degrade guar gum as well. On the other hand two strains of *B. distasonis* and *B. thetaiotaomicron* can ferment guar gum only after being treated by the aforesaid extracellular enzymes (Tomlin et al., 1986). It can be useful for the isolation and maintenance of thermophiles as it does not melt at temperatures as high as 70°C (Jain et al., 2005). Stable slants cannot be formed at room temperature with guar gum due to its high mobility and requires blending with other gelling agents (Jain, 2011). However, addition of cations or borax can improve the gelling properties of guar gum allowing it to be a suitable gelling agent for the growth of fungi and bacteria (Jain, 2011). Use of guar gum is restricted due to its inefficiency of self gellation and its high viscosity at higher temperature, which renders the dispensing of media to the petri plate difficult (Gangotri et al., 2012). It has less clarity due to the presence of impurities. In addition, due to its high adhesive property, it also hampers the isolation of microorganisms from a culture medium (Jain, 2011).

This non-ionic polysaccharide and its derivatives in various forms, such as coatings, matrix tablets, hydrogels and nanoparticles are often used in controlled release of therapeutics (Krishnaiah et al., 2002; Prabaharan, 2011; Aminabhavi and Nadagouda, 2014). In clinical trials, guar gum was reported to lower the serum cholesterol levels in patients with hypercholesterolaemia (Todd et al., 1990). Some other general applications of guar gum include paper sizing, thickener in syrups, protective colloid, stabilizer and as a binding and disintegrating agent in tablets. Its low cost makes it useful in plant tissue culture media (Babbar et al., 2005).

## Future directions

Several published alternatives of agar have not been studied very extensively for their properties. In fact, several physicochemical properties of different gums remain to be studied. Also the diversity of microorganisms that can be grown on them has been relatively unexplored. This might be achieved by using different gelling agents in combination than a single gelling agent. For example, a blend of Xanthan gum and agar in the ratio of 6:4 is recommended as an alternative to agar because of its suitability comparable to agar and cost advantage (Babbar and Jain, 2006). Alternative gelling agents like cassava flour, rice flour, corn flour, and potato starch in combination with agar is considered to be suitable for *in vitro* root regeneration (Daud et al., 2011) and we posit that they might have potential in microbial culture media too. Blending different gelling agents like guar gum, xanthan gum or isubgol with agar increases the viscosity and firmness of the media (Babbar and Jain, 2006).

There are several other sources that can be explored for gelling agents. We suspect that locust bean gum or carob gum, a galactomannan obtained from the seed endosperms of carob tree (*Ceratonia siliqua*), which has a wide range of applications in food (Ünal et al., 2003) and pharmaceutical industries (Brennan, 2005; Dionísio and Grenha, 2012) might find application as a gelling agent as well. Locust bean gum forms a viscous aqueous solution at relatively low concentration, which stabilizes emulsion and replaces fat in many food products. This unique characteristic makes it a very useful industrial gum. It is also non-ionic in nature and hence, solutions of locust bean gum are not influenced by pH, salts and heat treatment (Barak and Mudgil, 2014). Through its synergistic actions with hydrocolloids such as carrageenan and xanthan gum, locust bean gum forms a gel with more elasticity and strength (Tako and Nakamura, 1986; Copetti et al., 1997). It has also been reported that it supports the growth of pluripotent embryonic stem cells in an undifferentiated state in mouse which makes it viable and a non-animal derived alternative to other gels (Perestrelo et al., 2014). Katira gum, a colloidal polysaccharide obtained from the bark of *Cochlospermum religiosum* is used for plant cell culture (Jain and Babbar, 2002). It is transparent but its viscosity is significantly less than agar (Jain and Babbar, 2002), suggesting that it can be likely used in combination with agar. Welan gum, synthesized from *Sphingomonas* sp. (O'Neill et al., 1986), used in cement industry (Kaur et al., 2014), might also turn out to be a potential gelling agent. Starch sources from chickpea dextrose tapioca, corn, barley, potato and wheat have also been used as solidifiers for plant tissue culture (Sorvari, 1986a,b; Henderson and Kinnersley, 1988; Nene et al., 1996) but have not been explored as gelling agents in microbial media.

We hope for the speedy discovery of new gelling agents to cover the pH and temperature ranges that are not covered by the existing gelling agents. Work is required to find the reasons inhibition of certain microorganisms, if any, by the existing gelling agents and modifications to gelling agents to prevent their degradation by select group of microorganisms. Further research in the field is required to uncover newer insights of culturable microbes. In summary, there needs more progress in gelling agents to expand the range of culturable microbes and improve the quality of media.

### Conflict of interest statement

The authors declare that the research was conducted in the absence of any commercial or financial relationships that could be construed as a potential conflict of interest.
